# Supplementary material for: Shuterin Enhances the Cytotoxicity of the Natural Killer Leukemia Cell Line KHYG-1 by Increasing the Expression Levels of Granzyme B and IFN-γ through the MAPK and Ras/Raf Signaling Pathways
Source: Int J Mol Sci. 2022 Oct 24;23(21):12816. doi: 10.3390/ijms232112816 (PMC9654641; doi:10.3390/ijms232112816)
Supplement: Supplementary file 1 [file ijms-23-12816-s001.zip › ijms-1905616-supplementary.pdf]

Figure Supplement S1

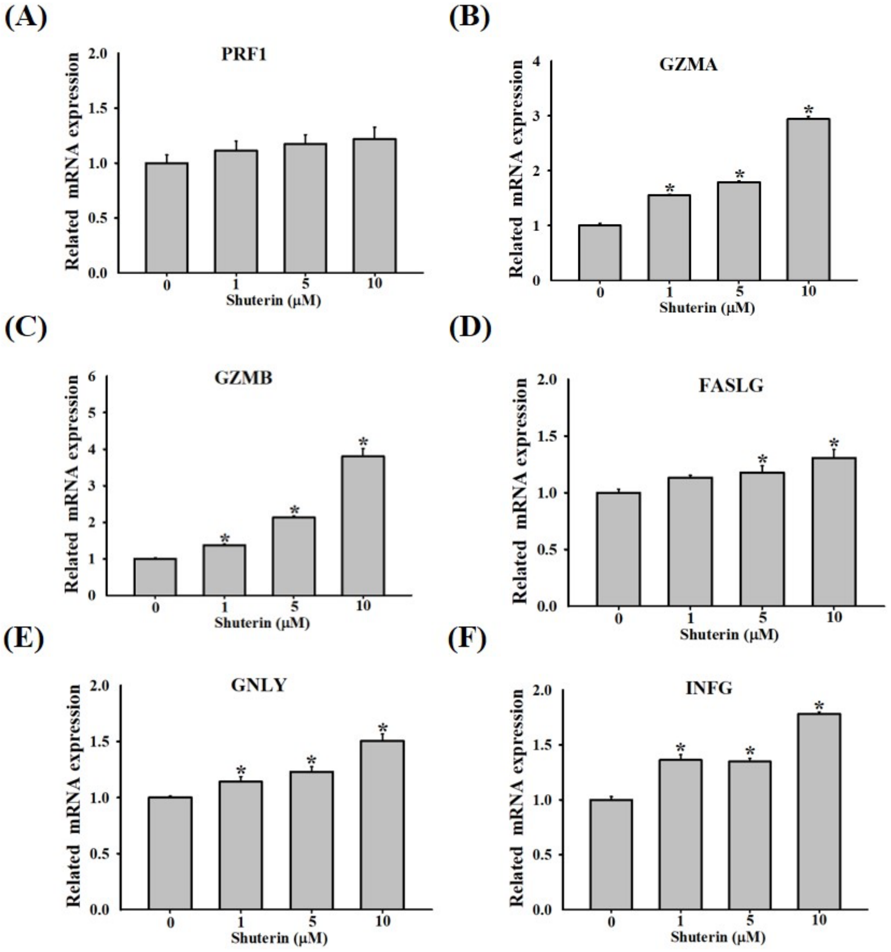

Figure S1. Effects of shuterin of the cytotoxic effector molecules on KHYG-1 cells. KHYG-1 cells were treated with shuterin for 24 h. The mRNA levels of Perforin (A), Granzyme A (B), Granzyme B (C), FasL (D), Granulysin (E), and IFN-γ (F) were determined by quantitative reverse transcription. The expression of each gene was normalized to the expression of GAPDH. Relative rations of gene expression V.S. control (assigned a value of 1) are indicated. Data are presented as mean ± standard deviation (n = 3). *$p < 0.05$ V.S. control.

Figure Supplement S2

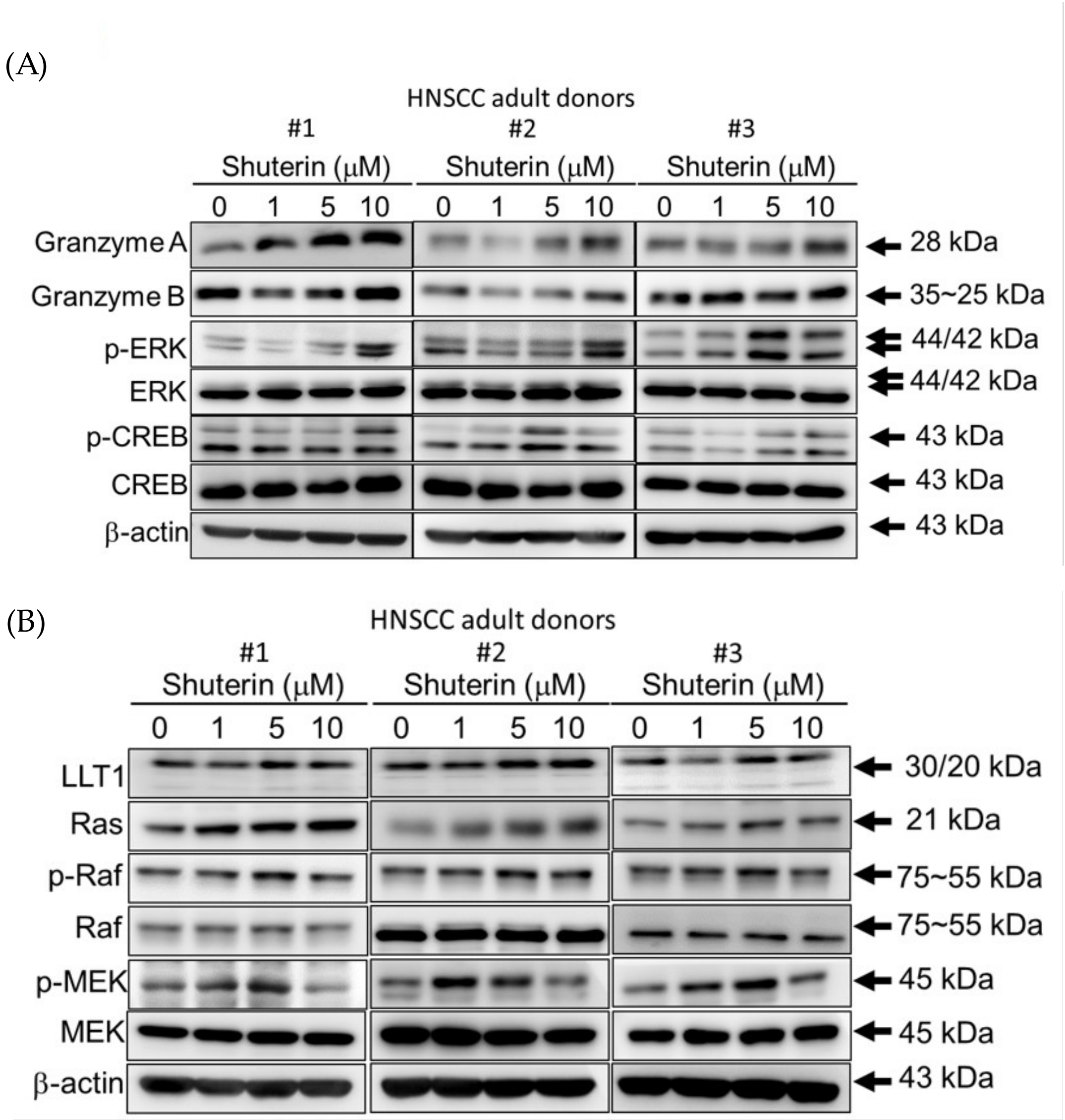

Figure S2. Effect of shuterin on protein levels of HNSCC adult donors of NK cells by Western blot analysis. NK cells from three different individual HNSCC adult donors were treated with the indicated doses of shuterin for 24 h. (A) Protein levels of cytotoxic effectors: granzyme A, granzyme B, p-EKR/ERK, and p-CREB/CREB. (B) Protein levels of LLT1 and Ras/Raf pathway (Ras, p-Raf, Raf, p-MEK, and MEK). Protein levels were determined using densitometry and were normalized to β-actin level. Data are presented as mean ± standard deviation (n = 3). *$p < 0.05$ V.S. control

Supplement Materials and Methods

1. Quantitative reverse transcription PCR (qPCR)

KHYG-1 cells were treated with shuterin (0, 1, 5, and 10 μM) for 24 h. Then the total RNA of samples was extracted using Zymo Research Quick-RNA™ Miniprep kit. RNA was reverse transcribed into cDNA using the High Capacity cDNA Reverse Transcription kit (Thermo Fisher Scientific, Inc.) according to the manufacturer's protocol. Subsequently, qPCR was performed using an ABI StepOne™ Real-Time PCR System (Applied Biosystems, Foster City, CA, USA), and analyzed with SetpOne Software vers. 2.3 (Applied Biosystems) using the TaqMan assay. Assay ID: Hs00989184_m1 (GZMA); Hs00188051_m1 (GZMB); Hs00169473_m1 (PRF1); Hs00246266_m1 (GNLY); Hs00989291_m1 (INFG); Hs00181225_m1 (FASLG). The all mRNA expression was quantified using the $2^{\Delta\Delta Cq}$ method and normalized to the internal reference gene GAPDH [1].

Reference:

[1] Livak KJ, Schmittgen TD. Analysis of relative gene expression data using real-time quantitative PCR and the 2(-Delta Delta C(T)) Method. Methods. 2001; 25: 402-8.
